# The Current Application Status of Pulmonary Function Testing in West China: A Cross‐Sectional Survey and Analysis

**DOI:** 10.1155/carj/7373734

**Published:** 2026-04-29

**Authors:** Naer An, Lijuan Gao, Jiangyue Qin, Weiguo Xu, Hailong Wei, Meng Li, Weiling Cai, Zhenni Chen, Lian Liu, Binmiao Liang, Yongchun Shen, Fuqiang Wen

**Affiliations:** ^1^ Department of Pulmonary and Critical Care Medicine, West China Hospital, Sichuan University, Chengdu, 610041, China, scu.edu.cn; ^2^ State Key Laboratory of Respiratory Health and Multimorbidity, West China Hospital, Sichuan University, Chengdu, 610041, China, scu.edu.cn; ^3^ Department of General Practice, West China Hospital, Sichuan University, Chengdu, 610041, China, scu.edu.cn; ^4^ Department of Pulmonary and Critical Care Medicine, Mianyang Central Hospital, Mianyang, 621000, China, myzxyy.com; ^5^ Department of Pulmonary and Critical Care Medicine, The People’s Hospital of Leshan, Leshan, 614000, China, leshan-hospital.com; ^6^ Department of Pulmonary and Critical Care Medicine, Liangshan Hospital of Integrated Traditional and Western Medicine, Xichang, 615050, China; ^7^ Department of Respiratory and Critical Care Medicine, Luojiang District People’s Hospital, Deyang, 618599, China; ^8^ Department of Medical Affairs, West China Hospital, Sichuan University, Chengdu 610041, China, scu.edu.cn

**Keywords:** pulmonary function testing, questionnaires, survey, West China

## Abstract

Pulmonary function testing (PFT) is a critical diagnostic and management tool for respiratory diseases, yet its implementation in China, particularly within primary hospitals, remains suboptimal. To investigate the current application status of PFTs specifically in western China, a cross‐sectional survey based on an online questionnaire was conducted. Physicians from various hospitals completed this questionnaire, designed according to Chinese PFT Guidelines, during 2023–2024, and the analysis focused on the final valid responses. The study ultimately included data from 488 hospitals. A total of 97.13% were public institutions. A total of 38.32% had established independent respiratory departments. The overall PFT availability was 56.76%, 100.00% in tertiary hospitals, and only 25.00% in primary hospitals. A total of 42.24% of the hospitals had a daily workload of less than 5 people. The primary tests included pulmonary ventilation tests (100.00%), pulmonary volume tests (73.65%), and bronchial dilation tests (72.92%). Instrument calibration was performed in 83.03% of the hospitals, and 68.59% of the hospitals considered filter resistance. Over 60.00% of hospitals used estimated values provided by instruments as sources for PFT. A total of 65.70% of hospitals had a limited number of operators. In conclusion, the application of PFT in western China is characterized by a low implementation rate, imbalanced distribution, limited test variety, and insufficient personnel, especially in primary hospitals. It is essential to promote the widespread implementation and standardization of PFTs in western China.

## 1. Introduction

Chronic respiratory diseases are the leading contributors to global morbidity and mortality, imposing significant burdens on both healthcare systems and society [1]. Chronic respiratory diseases rank as the third leading cause of death, with a prevalence of 454.6 million cases globally, and are responsible for more than 4.0 million deaths [2]. Countries with low‐ to middle‐income levels are at greater risk of death and disability caused by chronic respiratory diseases [3]. Chronic obstructive pulmonary disease (COPD) and asthma are the main disease types, and COPD is one of the major contributors to global mortality and incidence rates [4]. Asthma is the second leading cause of death from chronic respiratory diseases [5]. It is estimated that there are 99 million patients with COPD and 43.7 million patients with asthma in China, with prevalence rates of 13.70% and 4.30% among people aged 40 years or older, respectively [6, 7]. Enhancing early screening, comprehensive evaluation, and advanced implementation of preventive measures and therapies for chronic respiratory ailments under the Healthy China Initiative are highly important.

Pulmonary function testing (PFT) is a noninvasive diagnostic tool critical for evaluating lung function, with applications encompassing disease diagnosis, severity stratification, and therapeutic monitoring [8, 9]. With technological advancements and expanded test parameters, the scope of PFT continues to expand. Currently, it is extensively utilized in fields, such as respiratory medicine, surgery, anesthesia, epidemiology, diving, and aerospace medicine [10]. Global respiratory disease guidelines, such as the Global Initiative for Chronic Obstructive Lung Disease (GOLD), the Global Initiative for Asthma (GINA), Chinese clinical guidelines for COPD and asthma, and expert consensus on bronchiectasis diagnosis and treatment, emphasize the indispensable role of PFT in disease identification and evaluation. The Healthy China 2030 Action Plan prioritizes chronic respiratory disease prevention and management, recommending annual PFT for individuals aged ≥ 40 years or at high risk [11]. Despite policies promoting integrated health care, primary centers often face resource constraints and low PFT utilization. In western China, PFT integration into clinical and research settings remains understudied, particularly given unique demographic and environmental factors, such as high‐altitude populations and socioeconomic diversity.

Sichuan Province, located in western China, exemplifies a large, multiethnic province with imbalanced economic development. In 2017, its air quality index (AQI) averaged 4.41, ranking 13th among China’s 31 provinces [12]. The adult smoking rate in Sichuan is 30.34%, with 70.00% of nonsmokers exposed to second‐hand smoke [13]. A comparison of COPD excess mortality rates in four high‐PM_2.5_ regions (Beijing‐Tianjin‐Hebei, the Yangtze River Delta, the Pearl River Delta, and the Sichuan Basin) revealed that Sichuan presented the highest rate [14]. The significant chronic respiratory disease burden in Sichuan underscores the importance of early diagnosis. However, limited data exist on PFT utilization in Sichuan Province; ethnic diversity and geographic heterogeneity may influence lung function norms, thereby highlighting the necessity of understanding current PFT practices to optimize respiratory care. Therefore, this study examined challenges in the implementation of PFT across Sichuan Province hospitals to identify barriers and improvement strategies.

## 2. Methods

### 2.1. Survey Design and Collection

On the basis of the Chinese Guidelines for PFT [10, 15], we designed an online questionnaire for this survey from July 2023 to April 2024. The questionnaire comprises three sections: (1) Hospital Characteristics: level (primary/secondary/tertiary), type (public/private), establishment of an independent respiratory department (yes/no), number of beds, and physicians in the independent respiratory department; (2) PFT Application Status: implementation (yes/no), test types, duration, daily workload, source departments, routine examination use (yes/no), dedicated PFT room (yes/no), instruments, and operators; and (3) PFT Quality Control: calibration, medication, methods, frequency, expected value sources, diagnostic criteria, grading systems, and report content.

### 2.2. Data Collection

Hospitals in China are officially classified into three tiers by the National Health Commission (NHC) based on criteria including bed capacity, medical equipment, technical expertise, teaching mandates, and research output [15]. In this study, primary hospitals serve as the foundation of the healthcare system, delivering essential medical care, preventive services, and public health management. They are predominantly located in rural townships and urban residential communities. Secondary hospitals offer more comprehensive services, handling common and moderately complex diseases. They are typically situated in county seats and districts within smaller cities or larger urban centers. Tertiary hospitals represent the highest level of care, providing specialized, complex, and advanced medical services. They are primarily located in larger cities, including prefecture‐level cities and provincial capitals. The latest Sichuan Provincial Health Statistical Yearbook (2022) indicates that Sichuan Province contained 924 primary, 745 secondary, and 317 tertiary hospitals by the end of 2022 [16]. This study surveyed healthcare professionals involved in PFTs across all three levels within Sichuan Province. There are no comprehensive official records or data on the proportion of hospitals at each level that conduct PFT, so the response rates were calculated using the total number of hospitals at each level as the denominator. Participating physicians completed surveys via WeChat, and data collection was facilitated by Wenjuanxing (https://www.wjx.cn/).

### 2.3. Inclusion and Exclusion Criteria

The inclusion criterion was complete, accurate responses from Sichuan Province hospital physicians. The exclusion criteria were as follows: (1) specialty hospitals (e.g., psychiatric and stomatological); (2) incomplete/inaccurate questionnaires; and (3) duplicate submissions from single institutions (retained the most accurate response).

### 2.4. Statistical Methods

We analyzed the application of PFT across various hospitals, encompassing implementation, operation, and quality control. Descriptive statistics were used to summarize the baseline characteristics of participating hospitals and the implementation status of PFT. Categorical variables are presented as frequencies (percentages) and were compared across three hospital levels using the chi‐squared test. When overall tests were significant, post hoc pairwise comparisons were performed with Bonferroni correction (adjusted significance threshold: *α*′ = 0.017). All the data were analyzed via SPSS (Version 25.0), and the figures were generated using GraphPad Prism (Version 9.5).

## 3. Results

### 3.1. Baseline Information Regarding the Hospitals

**FIGURE 1:**
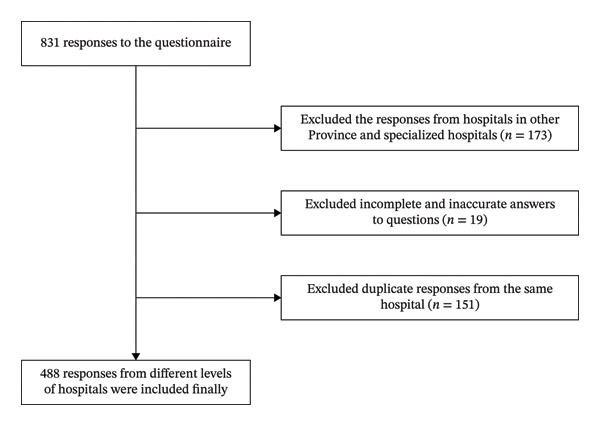
Flowchart of the screening process.

A total of 831 responses to the questionnaire were returned. After excluding 343 invalid responses (Figure 1), 488 hospitals from 18 prefecture‐level cities and 3 autonomous prefectures of Sichuan Province were ultimately included in this study. These hospitals comprised 260 primary hospitals (53.28%), 86 secondary hospitals (17.62%), and 142 tertiary hospitals (29.10%). Public hospitals predominated (474 hospitals, 97.13%), with 187 hospitals (38.32%) having established independent respiratory departments. Respiratory departments with 51–80 beds (51.87%) and 1–10 physicians (62.03%) accounted for the largest proportion. Detailed baseline characteristics of the enrolled hospitals are presented in Table 1.

**TABLE 1:** Baseline information of investigated hospitals in Sichuan Province.

Characteristic	*n* (%)
*Level of hospital (n = 488)*	
Primary	260 (53.28)
Secondary	86 (17.62)
Tertiary	142 (29.10)

Type of hospital (*n* = 488)	
Public	403 (97.13)
Private	14 (2.87)

Independent respiratory department (*n* = 488)	
Yes	187 (38.32)
No	230 (61.68)

Number of beds in the respiratory department (n = 187)*^∗^*	
≤ 50	22 (11.76)
51–80	97 (51.87)
81–120	42 (22.46)
121–150	14 (7.49)
> 150	12 (6.42)

Number of respiratory physicians (n = 187)*^∗^*	
1–10	116 (62.03)
11–20	47 (25.13)
21–30	15 (8.02)
31–40	4 (2.14)
41–50	0 (0.00)
> 50	5 (2.67)

*Note:* Data are presented as *n* (%). Primary hospitals are community health centers or township hospitals providing basic medical services. Secondary hospitals are regional hospitals providing comprehensive medical services. Tertiary hospitals are large, comprehensive, or specialized hospitals providing advanced medical care.

Abbreviation: PFT = pulmonary function testing.

^∗^ *n* = 187 represents hospitals with independent respiratory departments.

### 3.2. Implementation Status of PFT

Among the 488 hospitals, 277 hospitals (57.76%) implemented PFT, with a 100% implementation rate in tertiary hospitals and only 25% in primary hospitals. The primary barriers hindering PFT implementation were operator shortages (34.38%), instrument scarcity (33.68%), insufficient patients (20.49%), and limited PFT knowledge (11.46%). Among the hospitals conducting PFT, the principal projects included pulmonary ventilation tests (100%), pulmonary volume tests (73.65%), bronchial dilation tests (72.92%), diffusion function tests (54.51%), and residual gas volume measurements (54.15%). A limited number of hospitals, ranging from 5.05% to 17.33%, conducted bronchial provocation tests, forced oscillatory airway resistance tests, exercise cardiopulmonary function tests, plethysmography tests, and tide analysis.

### 3.3. Workload and Infrastructure of PFT

Most hospitals (58.85%) had less than 5 years of PFT experience. Daily workloads were reported as fewer than 5 persons/day in 42.24% of all hospitals and 95.38% of primary hospitals. Respiratory departments were the primary source for PFT (73.65%). Only 129 hospitals (46.57%) included PFT in routine physical examinations, and 197 hospitals (71.12%) had independent pulmonary function rooms. Both standard and portable instruments were available in 37.18% of hospitals, while most instruments were imported brands regardless of the hospital level. Disinfection awareness in all enrolled hospitals was adequately qualified. Details of the PFT implementation are shown in Table 2 and Figure 2.

**TABLE 2:** PFT implementation in hospitals in Sichuan Province.

Implementation of PFT	*n* (%)				*p*‐value
	Total (*n* = 488) (%)	Primary (*n* = 260) (%)	Secondary (*n* = 86) (%)	Tertiary (*n* = 142) (%)	
Implement	277 (56.76)	65 (25.00)^ac^	70 (81.40)^bc^	142 (100.00)^ab^	0.000
Projects					
Pulmonary ventilation test	277 (100.00)	65 (100.00))^ac^	70 (100.00)^bc^	142 (100.00)^ab^	0.000
Pulmonary volume test	204 (73.65)	23 (35.38)^ac^	56 (80.00)^bc^	125 (88.03)^ab^	0.000
Bronchial dilation test	202 (72.92)	10 (15.38))^ac^	59 (84.29)^bc^	133 (93.66)^ab^	0.000
Bronchial provocation test	48 (17.33)	1 (1.54)^ac^	8 (11.43)^bc^	39 (27.46)^ab^	0.000
Diffusion function test	151 (54.51)	4 (6.15))^ac^	34 (48.57)^bc^	113 (79.58)^ab^	0.000
Residual gas volume measurement	150 (54.15)	7 (10.77))^ac^	34 (48.57)^bc^	109 (76.76)^ab^	0.000
Peak expiratory flow rate check	116 (41.88)	10 (15.38))^ac^	23 (32.86)^bc^	83 (58.45)^ab^	0.000
Forced oscillatory airway resistance	37 (13.36)	0 (0.00))^ac^	4 (5.71)^bc^	33 (23.24)^ab^	0.000
Exercise cardiopulmonary function test	14 (5.05)	1 (1.54)^a^	1 (1.43)	12 (8.45)^a^	0.000
Plethysmography	23 (8.30)	1 (1.54)^a^	1 (1.43)^b^	21 (14.79)^ab^	0.000
Tide analysis	21 (7.58)	2 (3.08))^ac^	6 (857)^c^	13 (9.15)^a^	0.000
Development period (year)					0.000
< 1	11 (3.97)	7 (10.77)	2 (2.86)	2 (1.41)	
1–2	48 (17.33)	24 (36.92)	12 (17.14)	12 (8.45)	
3–5	104 (37.55)	33 (50.77))^ac^	36 (51.43)^bc^	35 (24.65)^ab^	
6–10	72 (25.99)	1 (1.54))^ac^	13 (18.57)^bc^	58 (40.85)^ab^	
11–20	34 (12.27)	0 (0.00))^ac^	6 (8.57)^bc^	28 (19.72)^ab^	
> 20	8 (2.89)	0 (0.00)^a^	1 (1.43)	7 (4.93)^a^	
Daily workload (person/day)					0.000
< 5	117 (42.24)	62 (95.38)^c^	32 (45.71)^bc^	23 (16.20)^b^	
5–10	90 (32.49)	3 (4.62))^ac^	29 (41.43)^c^	58 (40.85)^a^	
10–30	56 (20.22)	0 (0.00))^ac^	8 (11.43)^bc^	48 (33.80)^ab^	
30–60	11 (3.97)	0 (0.00)^a^	1 (1.43)	10 (7.04)^a^	
> 60	3 (1.08)	0 (0.00)	0 (0.00)	3 (2.11)	
Main source of patients					0.000
Respiratory department	204 (73.65)	12 (18.46))^ac^	59 (84.29)^c^	133 (93.66)^ab^	
Other internal medicine departments	14 (5.05)	10 (15.38)	3 (4.29)	1 (0.70)	
Cardiothoracic surgery	3 (1.08)	0 (0.00)	0 (0.00)	3 (2.11)	
Pediatrics	0 (0.00)	0 (0.00)	0 (0.00)	0 (0.00)	
Physical examination center	25 (9.03)	18 (27.69)^a^	5 (7.14)	2 (1.41)^a^	
Other	31 (11.19)	25 (38.46)^a^	3 (4.29)	3 (2.11)^a^	
Applied to routine physical examinations					0.000
Yes	129 (46.57)	12 (18.46))^ac^	32 (45.71)^bc^	85 (59.86)^ab^	
No	148 (53.43)	53 (81.54))^ac^	38 (54.29)^b^	57 (40.14)^a^	
Independent pulmonary function room					0.000
Yes	197 (71.12)	17 (26.15))^ac^	48 (68.57)^bc^	132 (92.96)^ab^	
No	80 (28.88)	48 (73.85)^a^	22 (31.43)^c^	10 (7.04)^ab^	
Instrument for PFT					0.000
Both standard and portable instruments	103 (37.18)	0 (0.00)^ac^	19 (27.14)^bc^	84 (59.15)^ab^	
Only standard instrument	46 (16.61)	0 (0.00))^ac^	15 (21.43)^c^	31 (21.83)^a^	
Only portable instrument	128 (46.21)	65 (100.00)^c^	36 (51.43)^bc^	27 (19.01)^b^	
Instrument brand					0.000
Imported	161 (58.12)	23 (35.38))^ac^	33 (47.14)^bc^	105 (73.94)^ab^	
Domestic	21 (7.58)	3 (4.62)^ac^	6 (8.57)^c^	12 (8.45)^a^	
Unclear	95 (34.30)	39 (60.00)^c^	31 (44.29)^bc^	25 (17.61)^b^	
Instrument disinfection					0.000
Sensor	208 (75.09)	48 (73.85)^ac^	50 (71.43)^bc^	110 (77.46)^b^	
Pipeline	170 (61.37)	31 (47.69)^ac^	44 (62.86)^c^	95 (66.90)^a^	
Elbow or handle	189 (68.23)	36 (55.38)^ac^	52 (74.29)^c^	101 (71.13)^a^	
Mask or mist storage tank	106 (38.27)	13 (20.00)^ac^	32 (45.71)^c^	61 (42.96)^a^	

*Note:* Data are presented as *n* (%).*p*‐values were calculated using the chi‐squared test. Post hoc pairwise comparisons were performed with Bonferroni’s correction (*α*′ = 0.017). Superscript letters indicate significant differences (*p* < 0.05) between groups.

Abbreviation: PFT = pulmonary function testing.

^a^significant difference in primary hospitals vs tertiary hospitals.

^b^significant difference in secondary hospitals vs. tertiary hospitals.

^c^significant difference in primary hospitals vs secondary hospitals.

FIGURE 2The PFT implementation in hospitals of different levels in Sichuan Province.

**Figure:**
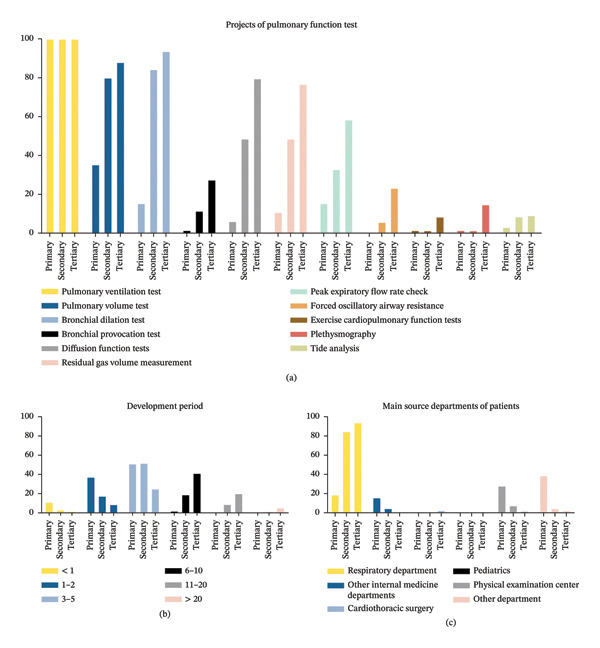

**Figure:**
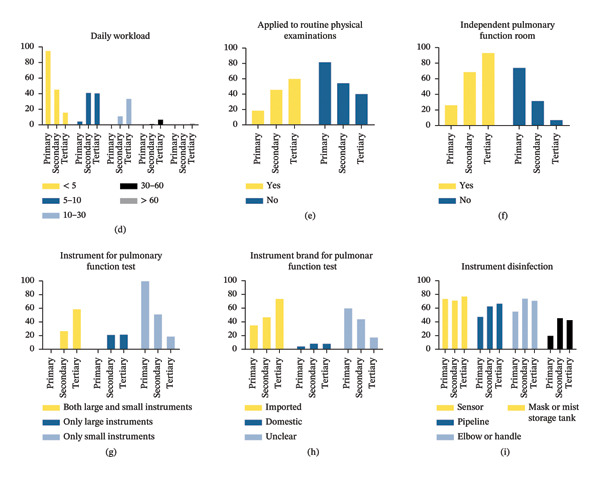

### 3.4. Operation and Quality Control of PFT

Among the 277 hospitals that conducted PFT, 230 hospitals (83.03%) recognized the necessity of calibration, and 190 hospitals (68.59%) considered filter resistance during the inspection process. A total of 202 hospitals (72.92%) relied on instrument‐derived estimated value as the source of PFT. For COPD diagnosis, 186 hospitals (67.15%) used the ratio of forced expiratory volume in the first second to forced vital capacity (FEV_1_/FVC) < 70% as the diagnostic threshold. A total of 165 hospitals (59.57%) used the five‐point method to classify pulmonary ventilation function test results. A total of 198 hospitals (71.48%) printed both flow and time–volume curves on the report forms. Details of the operation and quality control of PFT are outlined in Table 3.

**TABLE 3:** Operation and quality control of PFT in Sichuan Province.

Operational and quality control contents	*n* (%)
*Calibration of PFT (n = 277)*	
Yes	230 (83.03)
No	47 (16.97)

*Consider the resistance of respiratory filters (n = 277)*	
Yes	190 (68.59)
No	87 (31.41)

*Number of forced vital capacity test (n = 277)*	
1–2 times	136 (49.10)
3–8 times	122 (44.04)
> 8 times	19 (6.86)

*Expected value source for PFT (n = 277)*	
The instrument comes with an estimated value	202 (72.92)
Using correction factors	1 (0.36)
Chinese people’s own estimated value formula	40 (14.44)
Unclear	34 (12.27)

*F* *E* *V*_1_ */FVC judgment criteria for PFT (n = 277)*	
Actual measured value > 70%	186 (67.15)
Actual value/expected value% > 92%	85 (30.69)
Other	6 (2.17)

*Classification method for pulmonary ventilation function test results (n = 277)*	
Three‐point method (mild–moderate–severe)	106 (38.27)
Five‐point method (mild–moderate–moderately severe–severe–extremely severe)	165 (59.57)
Other	6 (2.17)

*Printing frequency of pulmonary ventilation function report form test data (n = 277)*	
1–2 times	203 (73.29)
3 times	65 (23.47)
> 3 times	9 (3.25)

*Print result graphics on pulmonary ventilation function report form (n = 277)*	
Flow–volume curve and time–volume curve	198 (71.48)
Flow–volume curve	32 (11.55)
Time–volume curve	2 (0.72)
No print result graphics	45 (16.25)

*Drugs used in bronchial dilation test (n = 202)*	
Salbutamol	198 (98.02)
Terbutaline	2 (0.99)
Isopropyl bromide	2 (0.99)

*Method of bronchial dilation test (n = 227)*	
Quantitative nebulization inhalation method	123 (54.19)
Dry powder inhalation method	89 (39.21)
Tidal breathing method	15 (6.61)

*Drugs used in bronchial provocation test (n = 48)*	
Histamine	5 (10.42)
Methacholine	32 (66.67)
Other	11 (22.92)

*Method of bronchial provocation test (n = 53)*	
Quantitative nebulization inhalation method	26 (49.06)
Hand pinch atomization inhalation method	8 (15.09)
2‐min tidal inhalation method	5 (9.43)
5 breaths method	14 (26.42)

*Method for diffusion function (n = 160)*	
One‐breath diffusion method	123 (76.88)
Internal breathing method	11 (6.88)
Repetitive breathing method	26 (16.25)

*Number of repetitions of diffusion function examination (n = 151)*	
< 2 times	70 (46.36)
2–5 times	80 (52.98)
> 5 times	1 (0.66)

*Note:* Data are presented as *n* (%). FEV1, forced expiratory volume in 1 s.

Abbreviations: FVC = forced vital capacity, PFT = pulmonary function testing.

### 3.5. Basic Information of Operators in PFT

Most hospitals (65.70%) have only 1–2 PFT operators. Over half of the operators held bachelor’s degrees (55.96%), and 73.65% of the hospitals provided more than 3 months of professional training. The operator characteristics are summarized in Table 4.

**TABLE 4:** Characteristics of PFT operators in hospitals in Sichuan Province.

Operators	*n* (%)				*P*‐value
	Total (*n* = 277)	Primary (*n* = 65)	Secondary (*n* = 70)	Tertiary (*n* = 142)	
Number					0.000
1–2	182 (65.70)	55 (84.62)^a^	51 (72.86)^b^	76 (53.52)^ab^	
3–5	81 (29.24)	7 (10.77)^a^	16 (22.86)^b^	58 (40.85)^ab^	
6–10	9 (3.25)	2 (3.08)	2 (2.86)	5 (3.52)	
> 10	5 (1.81)	1 (1.54)	1 (1.43)	3 (2.11)	
The highest degree					0.000
Below undergraduate	120 (43.32)	36 (55.38)^a^	35 (50.00)	49 (34.51)^a^	
Undergraduate	155 (55.96)	29 (44.62)^a^	35 (50.00)	91 (64.08)^a^	
Master	1 (0.36)	0 (0.00)	0 (0.00)	1 (0.70)	
Above master	1 (0.36)	0 (0.00)	0 (0.00)	1 (0.70)	
3 months of professional training					0.000
Yes	204 (73.65)	33 (50.77)^ac^	54 (77.14)^c^	117 (82.39)^a^	
No	73 (26.35)	32 (49.23)	16 (22.86)	25 (17.61)	

*Note:* Data are presented as *n* (%). *p*‐values were calculated using the chi‐squared test. Post hoc pairwise comparisons were performed with Bonferroni’s correction (*α*′ = 0.017). Superscript letters indicate significant differences (*p* < 0.05) between groups.

Abbreviation: PFT = pulmonary function testing.

^a^significant difference in primary hospitals vs tertiary hospitals.

^b^significant difference in secondary hospitals vs. tertiary hospitals.

^c^significant difference in primary hospitals vs secondary hospitals.

## 4. Discussion

PFTs are crucial for diagnosing respiratory diseases, assessing disease severity, evaluating therapeutic efficacy, and determining prognosis. This survey, conducted in Sichuan Province, identified a suboptimal PFT implementation rate of 56.76%. Tertiary hospitals achieved complete implementation, in contrast to the limited 25% rate in primary hospitals. Hospitals performing PFT demonstrated limited projects and low daily workloads, insufficient standardized equipment and trained operators, and deficient examination protocols and quality control.

PFT implementation in China remains suboptimal. A multiprovince survey of 212 tertiary hospitals in 2002 reported a 91.90% PFT utilization rate [17]. Sichuan Province has significantly lower implementation rates compared to the national average, especially in primary hospitals. A study on the correlation between pulmonary ventilatory function and cognitive function in southwestern China revealed a high prevalence of obstructive ventilatory dysfunction among middle‐aged and elderly individuals in that region. However, PFT has not yet been systematically incorporated into routine health management [18]. As a cornerstone of healthcare systems, primary care provides initial diagnoses for most chronic respiratory diseases [19]. The underutilization of PFT in primary care settings frequently leads to underdiagnosis and misdiagnosis of chronic respiratory disease [20]. However, only 9.70% of Chinese adults have received PFT, only 12% of COPD patients have undergone prior testing, and only 23.40% of asthma patients have received PFT [6, 21]. Additionally, primary hospitals reported 22.90% misdiagnosis rates and 22.10% missed diagnosis rates for COPD [22]. Consistent with these findings, 95.38% of the primary hospitals in this study reported < 5 persons/day workloads, indicating underutilization despite equipment and operator availability.

Research indicates that over 70% of primary healthcare facilities are actively engaged in various aspects of disease management for diabetes and hypertension, including disease screening, health record documentation, follow‐up services, health education, and professional training for healthcare providers. In contrast, the management of chronic respiratory diseases in primary hospitals is inadequate in all dimensions, with a proportion of less than 15% [23]. Therefore, standardizing the diagnosis and treatment of chronic respiratory diseases in primary care is imperative. A fundamental understanding of PFT in the diagnostic process should be established, PFT should be actively conducted for high‐risk populations, initial diagnosis rates of COPD and other chronic respiratory ailments in primary healthcare settings need to be improved, and the optimal timing for disease management needs to be improved.

Widespread implementation of easy PFT projects can significantly decrease the rates of missed diagnoses and misdiagnoses of respiratory conditions. With a 100% implementation rate, the pulmonary ventilation test is the most frequently applied and clinically significant PFT project regardless of hospital level, which reflects changes in lung volume, airway patency, and thoracic and respiratory muscle function, assisting in the diagnosis of chronic respiratory diseases, such as COPD [24]. Asthma, a prevalent chronic airway disease and major public health challenge in China, affects 45.7 million adults nationally [22]. The bronchial provocation test has been recognized as one of the gold standards in asthma diagnosis to ascertain airway obstruction and its variability, particularly in identifying cough variant asthma (CVA) [23]. However, only a limited number of hospitals conduct bronchial provocation tests. Only 28.80% of asthma patients receive diagnoses, and only 23.40% undergo prior PFT, indicating limited disease awareness and PFT access, especially in underdeveloped regions of China [25]. This limitation may also stem from the unavailability of certified provocative agents. Histamine and methacholine have similar bronchoconstrictive effects at equivalent dosages, but histamine is associated with increased systemic side effects and poor reproducibility [26]. However, histamine remains widely used because of its lower cost and established methodology. Given the clinical value of bronchial provocation testing, establishing certified provocative agents in China is urgently needed.

Inadequate standardization constitutes a major contributor to diagnostic inconsistency in PFT. The critical priority lies in maintaining quality control and enhancing diagnostic consistency through implementing unified standards and integrating diverse testing methodologies [27]. Our investigation indicates that some Chinese hospitals fail to adhere to PFT standards, particularly with respect to quality control, reporting content, and interpretation. Both the American Thoracic Society (ATS) and the European Respiratory Society (ERS) emphasize the necessity of calibration and specialized filter use [28]. Although filter use increases airflow resistance and dead space volume, factors potentially bias PFT parameters and require test‐specific consideration [29]. Nevertheless, many hospitals neglect both requirements. Most imported instruments contain reference equations potentially unsuitable for Chinese populations, yet hospitals predominantly rely on manufacturer‐provided values. Zheng and Zhong proposed correction factors for local populations [30], but only one surveyed hospital implemented this approach.

FEV_1_/FVC is a key diagnostic indicator for airway obstruction and COPD diagnosis. The lower limit of normal (LLN) represents the 95% confidence interval cut‐off for abnormal results [8]. Chinese guidelines recommend an FEV_1_/FVC ≥ 92% of the predicted value as the threshold for simplicity [17]. However, only 30.69% of hospitals have adopted this method, whereas 67.15% have erroneously applied the COPD diagnostic criterion (FEV_1_/FVC < 70%), risking age‐related misdiagnosis and overlooking early‐stage patients [31, 32]. Guidelines recommend classifying ventilatory impairment severity via the FEV_1_% predicted via five‐point grading [33]. However, 38.27% still used three‐point grading. Inconsistent report formats and variable indicator expressions risk misinterpretation, hinder data sharing, and challenge future PFT big data initiatives. Substantial improvement in PFT standardization is needed.

Over half of the tertiary hospitals possessed both standard and portable pulmonary function instruments, whereas primary hospitals predominantly relied mainly on portable instruments. Portable instrument affordability, operational ease, and portability render them ideal for primary hospitals [34]. Furthermore, essential parameters, such as the FEV_1_, FVC, and peak expiratory flow rate (PEF), demonstrate no significant differences compared to those of standard instruments, aligning well with primary‐level routine testing requirements [35]. Therefore, enhancing the availability and standardized utilization of portable PFT instruments in primary hospitals, particularly in rural and economically disadvantaged regions, is crucial [36]. Among the investigated hospitals, 58.12% were equipped with imported PFT instruments, and 72.92% relied solely on device‐provided estimated values. This underscores the urgent need for relevant departments to advance domestic instrument development and quality. Affordable, effective domestic instruments would significantly improve primary hospitals’ PFT services.

Primary care institutions in China face human resource shortages. Additionally, the inherent complexity of PFT imposes higher demands on primary care personnel [37], and operators performing PFTs must ensure device accuracy and precision [38]. Moreover, biases exist in quality control systems across different institutions due to varying proportions of qualified personnel and technical variations, necessitating resolution through standardized training [39]. This reflects the inadequate implementation of quality control protocols and underscores the critical need for professional training. In this study, personnel shortages persisted across all hospital levels, with most facilities employing only 1–2 operators in charge, and only 73.65% of operators received professional training. Operator expertise is vital for advancing respiratory and critical care, as well as the comprehensive prevention and treatment of respiratory diseases. It is essential to strengthen the allocation of dedicated personnel for PFT; provide specialized knowledge lectures and continuing education courses through medical alliances, specialty alliances, remote health care, and other channels; and enhance training and assessment in operational techniques, quality control, and diagnostic report interpretation [36].

As a comprehensive PFT survey across Sichuan Province’s multilevel healthcare facilities, this study provides foundational data for chronic respiratory disease big data platforms, potentially increasing the diagnostic and treatment standards for such conditions in Sichuan Province. However, there are certain limitations in this study. First, the sample size was relatively limited, as the response rates varied across hospital tiers, particularly in primary hospitals, which exhibited lower participation rates. This limited the representativeness of this study’s findings across the entire province of Sichuan. Second, the study failed to adhere to the scientific requirements of random sampling to adequately stratify hospitals by tier, which could introduce selection bias. Third, the study spanned a relatively long period, during which some hospitals may have introduced or upgraded PFT equipment, potentially leading to biases in the study results. Finally, the absence of systematic data on PFT infrastructure in Sichuan Province represents a critical gap in interpreting our findings. This study provides a snapshot of PFT coverage across hospitals at each level. Future studies should prioritize the collection and reporting of PFT infrastructure data to enable more accurate assessments of the availability and equity of diagnostic services. Additionally, efforts should focus on expanding the survey scope to capture a more comprehensive provincial insight into lung function development; employing stratified random sampling with enhanced coverage in different levels of hospitals to strengthen representativeness; and implementing strict temporal controls or dynamic monitoring to mitigate biases from equipment upgrades during the study period.

## 5. Conclusion

In summary, PFT utilization in West China has demonstrated suboptimal implementation, uneven resource allocation, limited project availability, and insufficient personnel, particularly in primary hospitals. Standardization of quality control, reporting formats, and interpretation requires urgent improvement.

## Author Contributions

Naer An, Lijuan Gao, and Jiangyue Qin contributed equally to this work and share first authorship. Naer An, Lijuan Gao, and Jiangyue Qin: conception, design, materials, analysis and/or interpretation, literature review, and writing. Weiguo Xu, Hailong Wei, Meng Li, Weiling Cai, Zhenni Chen, and Lian Liu: materials, data collection, and/or processing. Binmiao Liang: conception, supervision, and critical review. Yongchun Shen: conception, supervision, fundings, and critical review. Fuqiang Wen: supervision and critical review.

## Funding

This work was supported by the Program from Science and Technology Department of Sichuan Province (grant numbers 2024YFFK0279, 2024NSFSC1522, and 2025ZNSFSC1543), the National Natural Science Foundation of China (grant numbers W2511097, 82170046, and 82300050), grant from 1·3·5 Project of State Key Laboratory of Respiratory Health and Multimorbidity, West China Hospital, Sichuan University (grant number RHM24207), and 1•3•5 Project for Disciplines of Excellence, West China Hospital of Sichuan University (grant number ZYGD23009), and the Science and Technology Projects of Xizang Autonomous Region, China (grant number XZ202501ZY0120).

## Disclosure

All authors read and approved the final manuscript. The funders had no role in study design, data collection and analysis, decision to publish, or preparation of the manuscript.

## Ethics Statement

This study used a completely anonymous questionnaire to investigate the application of lung function in Sichuan Province. The questionnaire design does not collect any personal identification information, ensuring that data collection and analysis are conducted at a completely anonymous aggregation level. Therefore, ethical review and informed consent are not applicable.

## Conflicts of Interest

The authors declare no conflicts of interest.

## Data Availability

The data that support the findings of this study are available upon request from the corresponding author. The data are not publicly available due to privacy or ethical restrictions.
